# Supplementary material for: Spatial Segregation Within Dissolving Microneedle Patches Overcomes Antigenic Interference and Enables Potent Bivalent Influenza–RSV Vaccination in Mice
Source: Vaccines (Basel). 2025 Nov 30;13(12):1213. doi: 10.3390/vaccines13121213 (PMC12737591; doi:10.3390/vaccines13121213)
Supplement: Supplementary file 1 [file vaccines-13-01213-s001.zip › vaccines-3982411-supplementary.pdf]

SUPPLEMENTAL FIGURES

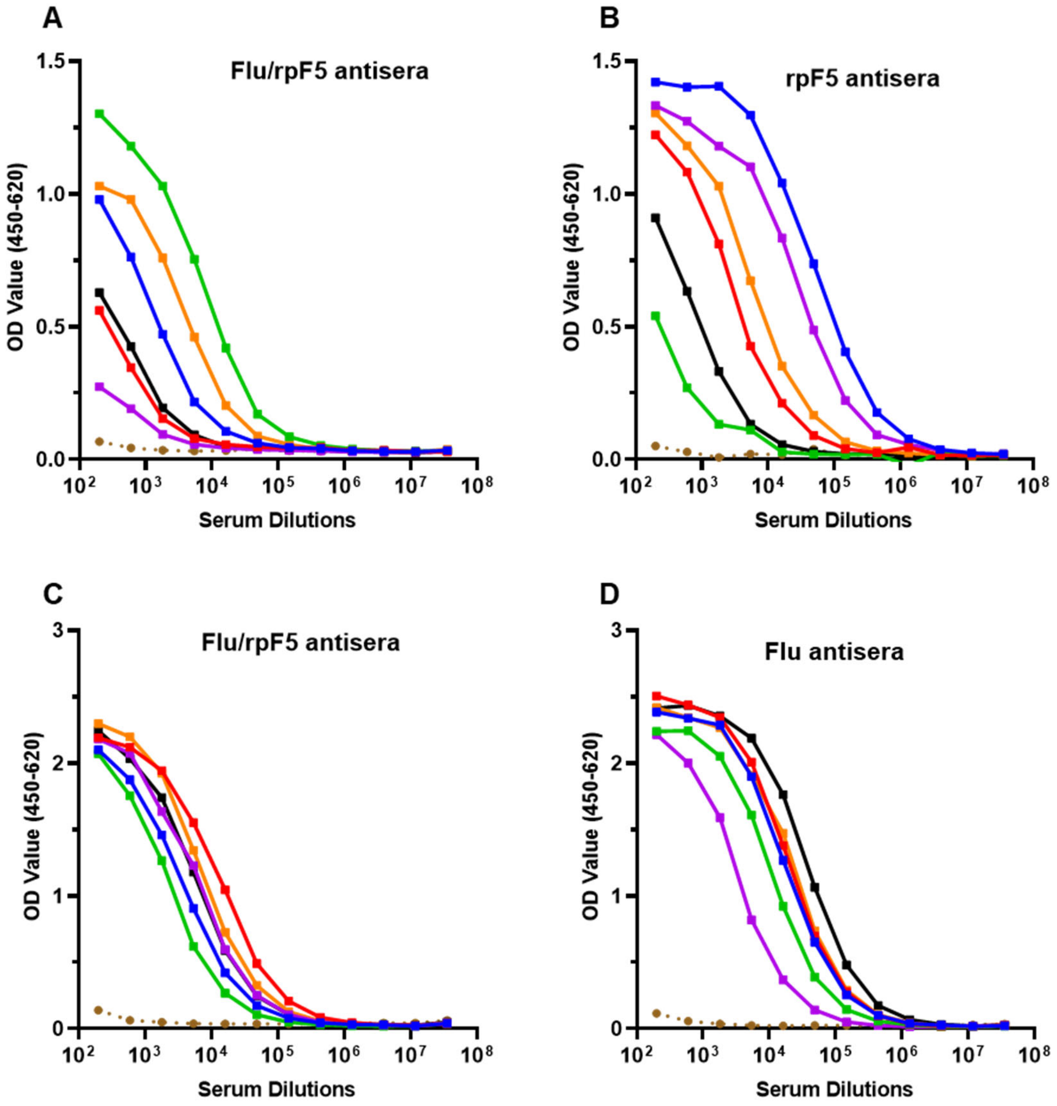

Figure S1 IgG responses induced by Flu A/Darwin split-virus, rpF5 or H3N2/rpF5 vaccination in mice. Serum samples from groups BALB/c mice (n=6) i.m. immunized twice with alum-adjuvanted rpF5 (10µg/dose), or Flu A/Darwin split-virus (1.5 µg/dose, without adjuvant), or premixed Flu A/Darwin split-virus and rpF5 (1.5 µg Flu + 10µg preF5/dose) were individually titrated, in triplicate wells, against recombinant rpF5 (**A, B**) or Flu A/Darwin virus (**C, D**) in ELISAs for antigen-specific IgG Abs. Normal mouse serum (NC) was included as controls. The data are mean absorbance at OD450-620 of each biological sample.

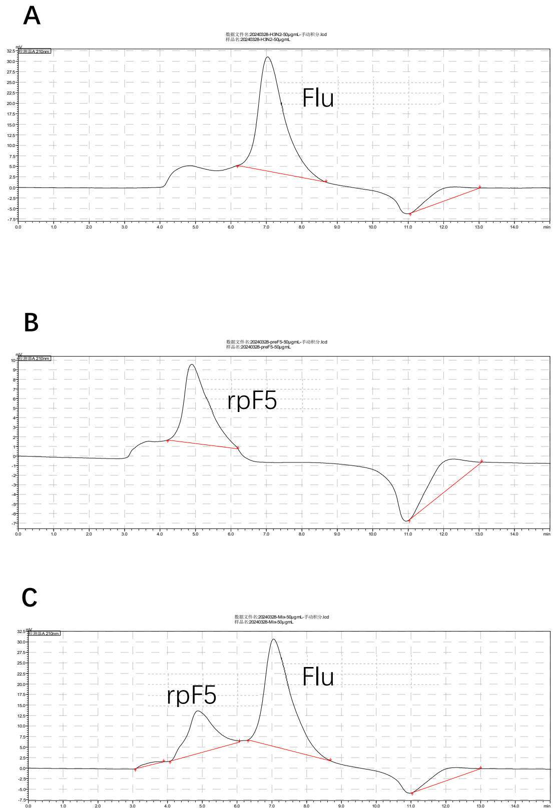

Figure S2 HPLC profiles of the H3N2 and rpF5 and H3/RSV combo vaccines. HPLC profiles of the Flu A/Darwin split-virus (**A**), rpF5 (**B**) and mixture of the two (**C**) are shown. Chromatography Column: Sepax Zenix-C SEC-300; Mobile Phase: water (100 mM PB (Phosphate Buffer), 100 mM Na_2SO_4), pH 6.7; Elution Program: Isocratic elution at 1.0 mL/min; Column Temperature: 25°C; Detection Wavelength: 280 nm

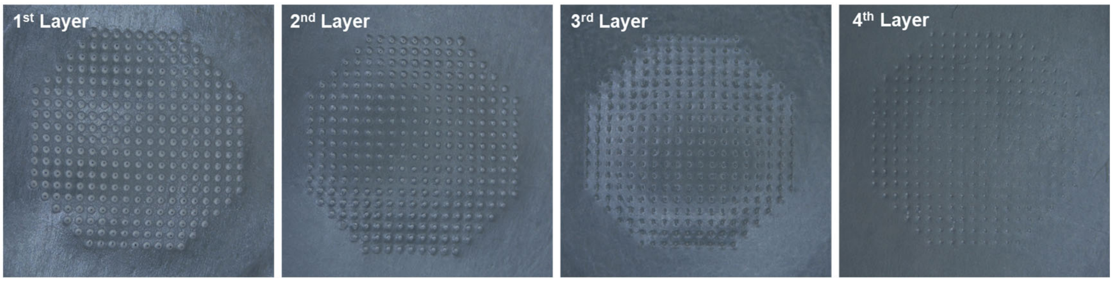

Figure S3 Mechanical integrity and penetration capability of the fabricated D-MAP patches. The strength of D-MAP-based combo-vaccines was checked before and after short-term insertion in a ten-layer parafilm pack for penetration effectiveness, bending and brittleness under the microscope. Photographs show penetration imprints on the top 4 layers of parafilm pack caused by a MAP-Flu/RSV patch.

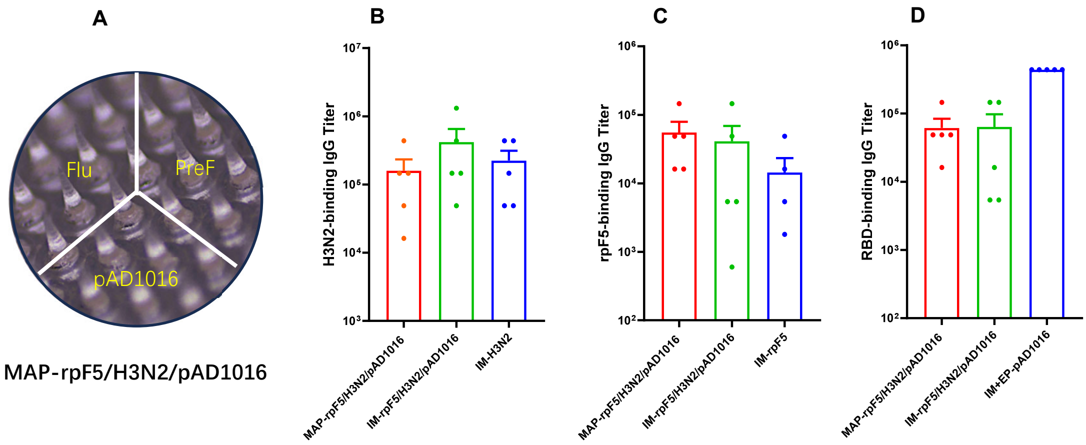

Figure S4 Immunogenicity of a D-MAP-based Flu-RSV-COVID-19 combo-vaccine. (**A**) Micrograph of a D-MAP partition-loaded with 1.5 µg Flu A/Darwin split-virus, 10 µg rpF5 and 20 µg DNA plasmid pAD1016 encoding heterodimeric RBD of SARS-CoV-1 and SARS-CoV-2 (MAP-rpF5/H3N2/pAD1016). (**B-D**) BALB/c mice (n=5) were vaccinated twice, on Days 0 and 14, with either MAP-rpF5/H3N2/pAD1016, or IM-delivered mixture of rpF5, H3N2 Flu split-virus and pAD1016 plasmid (assisted with electrophoresis), or standalone Flu A/Darwin split-virus (1.5 µg/dose), or rpF5 (10µg/dose, alum adjuvanted), or pAD1016 plasmid (20 µg/dose, assisted with electrophoresis). Serum samples, collected 15 d after the boost immunization, were titrated against the Flu split-virus (**B**), rpF5 (**C**) or recombinant RBD of wildtype SARS-CoV2 (**D**) in ELISAs. Data are mean ± SEM of serum IgG titers.

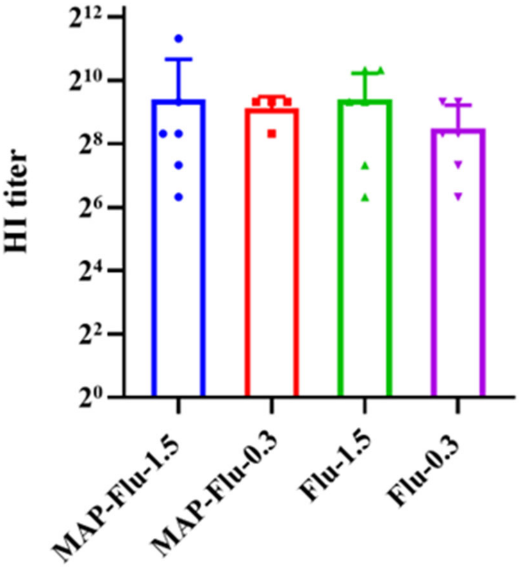

Figure S5 Hemagglutinating serum Ab responses induced by Flu antigen immunization in mice. BALB/c mice (n=6) were vaccinated twice, on Days 0 and 14, with i.m. administered Flu H3N2 antigen (0.3 µg or 1.5 µg/dose), or D-MAPs carrying 0.3 µg (MAP-Flu-0.3), or 1.5 µg (MAP-Flu-1.5), Flu H3N2 antigen. Serum samples, collected on day 28, were titrated in HI assays against A/Victoria virus. Data are mean ± SEM of log2 HI titers.

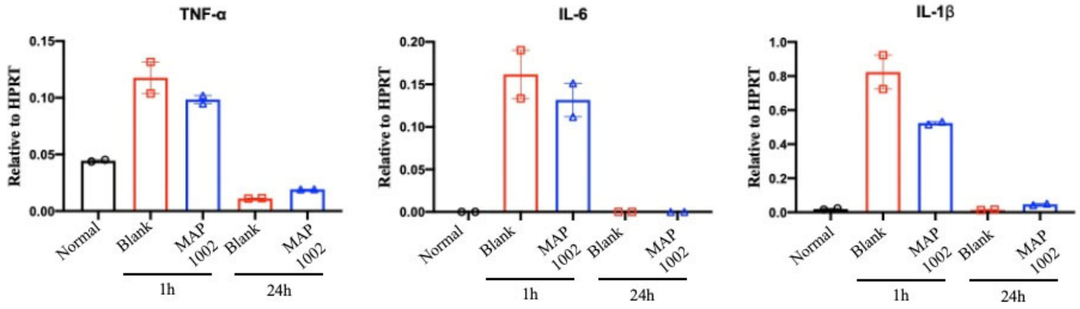

Figure S6 Immediate inflammatory cytokine responses of local skin tissue following D-MAP application. Groups of BALB/c mice (n=3) were treated for 15 min with D-MAPs laden with plasmid pAD1002 or D-MAP-placebo (Blank), and sacrificed 1 or 24 h later for their MAP-treated skin sites which were subjected to RNA extraction and Q-PCR detection of TNFα, IL-6 and IL-1β mRNA transcription. Untreated normal mouse skin (Normal) was included as additional control. The results are expressed relative mRNA expression compared to HPRT.

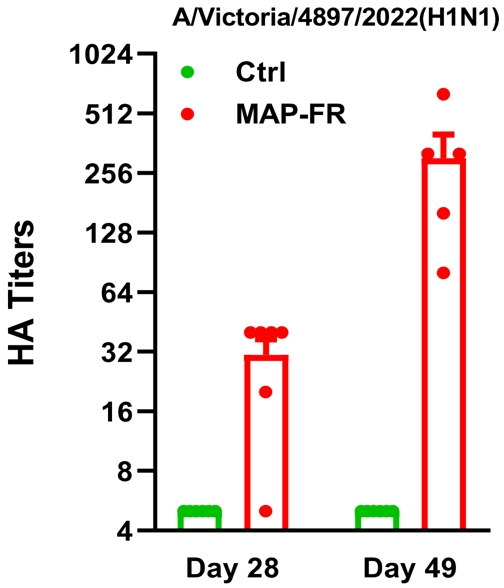

Figure S7 Neutralizing Ab responses in BALB/c mice following MAP-Flu/RSV vaccination. BALB/c mice (n=5) were vaccinated twice, on Days 0 and 28, with partition-loaded MAP-Flu/RSV or MAP-placebo (Ctrl). Serum samples, collected on days 28 and 49, were titrated HI assays against A/Victoria virus. Data are mean ± SEM of HI titers.
